# Feasibility of endoscopic ultrasound-guided hepaticogastrostomy using a 22-gauge needle

**DOI:** 10.1097/MD.0000000000031545

**Published:** 2022-11-04

**Authors:** Koji Takahashi, Hiroshi Ohyama, Mayu Ouchi, Motoyasu Kan, Hiroki Nagashima, Yotaro Iino, Yuko Kusakabe, Kohichiroh Okitsu, Izumi Ohno, Yuichi Takiguchi, Naoya Kato

**Affiliations:** a Department of Gastroenterology, Graduate School of Medicine, Chiba University, Chiba, Japan; b Department of Medical Oncology, Graduate School of Medicine, Chiba University, Chiba, Japan.

**Keywords:** endoscopic ultrasound, guidewire, hepaticogastrostomy, stent

## Abstract

This study aimed to evaluate the feasibility of performing endoscopic ultrasound-guided hepaticogastrostomy using a 22-gauge fine-needle aspiration needle. This was a single-center retrospective study. Fourteen patients who underwent endoscopic ultrasound-guided hepaticogastrostomy with a 22-gauge fine-needle aspiration needle were examined. Fourteen eligible patients were included in this study. The age of patients ranged from 55 to 93 years, with a median of 76 years. Of patients with existing underlying diseases, there were 8 cases of pancreatic cancer (57.1%), 2 cases of metastatic liver tumor (14.3%), 2 cases of bile duct stones (14.3%), 1 case of hilar cholangiocarcinoma (7.1%), and 1 case of gallbladder cancer (7.1%). Regarding gastrointestinal anatomy, there were 11 cases (78.6%) of normal and 3 cases (21.4%) of gastric resection with Roux-en-Y. Reasons for endoscopic ultrasound-guided hepaticogastrostomy were duodenal obstruction in 7 cases (50.0%), surgically altered anatomy in 3 cases (21.4%), and 4 cases (28.6%) of failed endoscopic retrograde cholangiopancreatography. Technical success was achieved in 11 cases (78.6%). Subsequently, 11 cases of technical success were analyzed. There were 5 cases of puncturing B2 (45.5%). The puncture bile duct diameter ranged from 3.1 to 5.7 mm, with a median of 4.4 mm. endoscopic ultrasound-guided antegrade procedures was combined with endoscopic ultrasound-guided hepaticogastrostomy in 2 cases (18.2%). Clinical success was achieved in all the cases. The procedure time ranged from 15 to 93 minutes, with a median duration of 35 minutes. Regarding the type of stent placed in hepaticogastrostomy, a plastic stent was placed in 10 cases (90.9%) and a metal stent was placed in 1 case (9.1%). Early adverse events occurred in 4 cases (36.4%), and all of these cases developed biliary peritonitis, late adverse events occurred in 1 case (9.1%), this was biloma. A change to a 0.025-inch guidewire during the procedure was required in 8 cases (72.7%). Esophageal puncture was not performed. endoscopic ultrasound-guided hepaticogastrostomy using a 22-gauge fine-needle aspiration needle is effective. However, in 72.7% of the cases started using the 0.018-inch guidewire, the guidewire was exchanged for a 0.025-inch guidewire during procedure.

## 1. Introduction

Biliary stenting using endoscopic retrograde cholangiopancreatography (ERCP) is the standard biliary drainage procedure for malignant biliary obstructions. However, in cases when ERCP fails or duodenal obstruction or surgically altered anatomy occurs, endoscopic ultrasound-guided biliary drainage (EUS-BD) is performed.^[1–3]^ The first case report on EUS-BD was published in 2001.^[4]^ EUS-BD includes 4 major techniques: EUS-guided choledochoduodenostomy, endoscopic ultrasound-guided hepaticogastrostomy (EUS-HGS), © endoscopic ultrasound-guided antegrade procedures (EUS-AG), and EUS-guided rendezvous technique. In EUS-HGS, a combination of a 19-gauge fine-needle aspiration (FNA) needle and 0.025-inch guidewire is generally used as a 0.025-inch guidewire provides better support for device insertion than a 0.018-inch guidewire. However, for patients with insufficient intrahepatic bile duct dilation, puncture with a 19-gauge FNA needle is challenging. In such cases, EUS-HGS is performed with a 22-gauge FNA needle and 0.018-inch guidewire.^[5,6]^ Using a 22-gauge FNA needle rather than a 19-gauge FNA needle, it is possible to puncture a small diameter bile duct. This makes it possible to perform EUS-HGS, even in cases where it cannot be performed with a 19-gauge FNA needle. However, when using a 22-gauge FNA needle, only a 0.018-inch guidewire could be inserted into the needle. Force transmission of the 0.018-inch guidewire was inferior to that of the 0.025-inch guidewire. Existing evidence regarding the use of a 22-gauge FNA needle with a 0.018-inch guidewire in EUS-HGS is limited. There are several steps of EUS-HGS including puncturing the intrahepatic bile duct from the stomach, dilation of the fistula, and plastic stent placement.^[7]^ This study aimed to evaluate the feasibility and safety of using a 22-gauge FNA needle compared to a 19-gauge FNA needle for EUS-HGS.

## 2. Patients and Methods

### 2.1. Study design

This was a single-center retrospective study. This study included patients who initially underwent EUS-HGS using a 22-gauge FNA needle combined with a 0.018-inch needle at our hospital between November 2019 and April 2022. The baseline characteristics and clinical outcomes of eligible patients were retrospectively examined from the medical records. Baseline characteristics included age, sex, underlying disease, gastrointestinal anatomy, and purpose of the procedure. Clinical outcomes included puncture site, diameter of the punctured bile duct, rate of combination with EUS-AG, clinical success rate, procedure time, type of stent placement for hepaticogastrostomy, rate of both early and late adverse events, and incidence of esophagus puncture.

Written informed consent for the procedure was obtained from all patients. This study was approved by the ethics committee of our hospital. Consent for patient participation in this study was obtained using opt-out methodology. This study was conducted in accordance with the principles of the Declaration of Helsinki.

### 2.2. Definitions

Clinical success and adverse events were defined according to the Tokyo Criteria 2014.^[8]^ Clinical success was defined as a decrease in the serum total bilirubin level to <50% or <2.0 mg/dL within 14 days of stent placement without additional biliary treatments. Procedure time was defined as the time interval between insertion of the endoscope from the mouth and its removal. Early and late adverse events were defined as procedure-related adverse events that occurred within 30 days of stent placement.

### 2.3. Techniques

**Figure 1.**
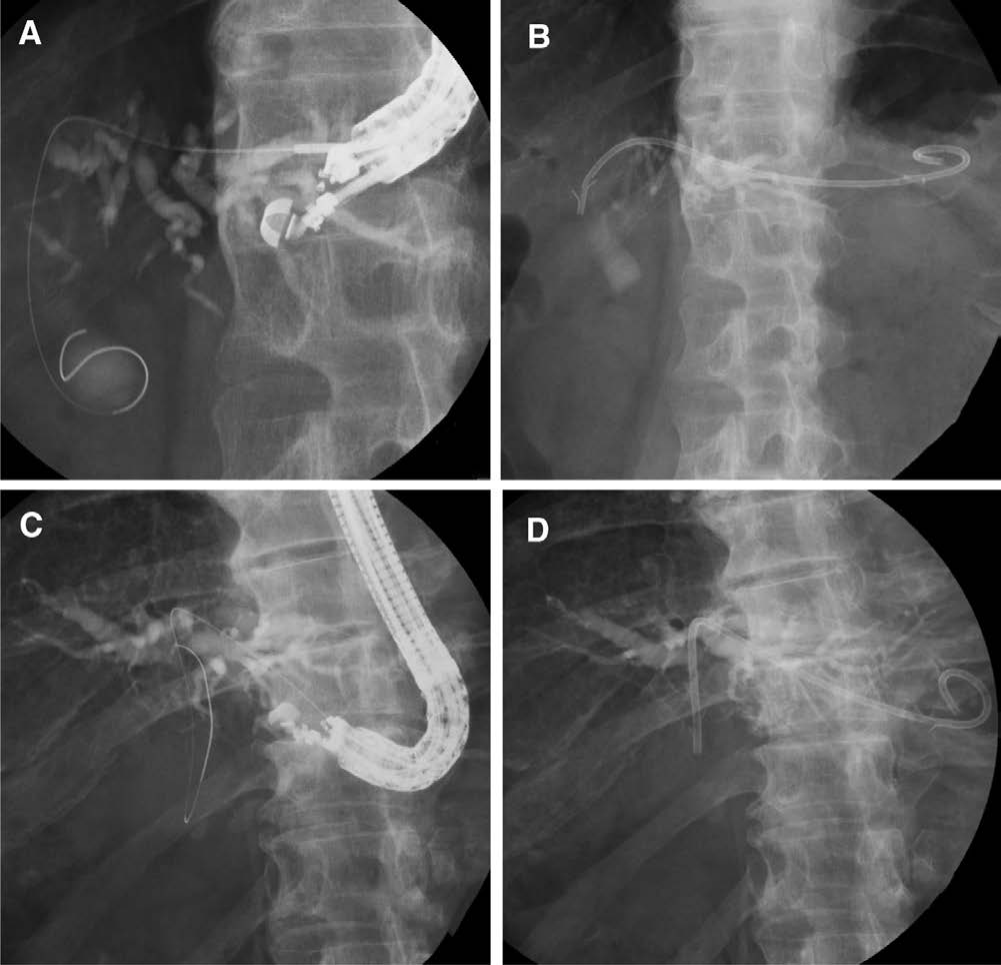
Fluoroscopic images of endoscopic ultrasound-guided hepaticogastrostomy performed using (A and B) a 22-gauge FNA needle combined with a 0.018-inch guidewire and using (C and D) a 19-gauge FNA needle combined with a 0.025-inch guidewire. The visibility of the 0.018-inch guidewire is almost the same as that of the 0.025-inch guidewire. FNA = fine-needle aspiration.

Prior to EUS-HGS, antibiotics were administered intravenously. EUS-HGS was performed by using an oblique-viewing linear echoendoscope (GF-UCT260; Olympus, Tokyo, Japan). Carbon dioxide insufflation was used during the procedure unless contraindicated. The intrahepatic bile duct from the stomach was identified and punctured using a FNA needle (EZ Shot 3 Plus; Olympus, Tokyo, Japan) under EUS guidance. Sodium meglumine amidotrizoate contrast medium was injected, and cholangiography was performed. A 0.018-inch guidewire (NovaGold; Boston Scientific, Tokyo, Japan or Fielder 18; ASAHI INTECC, Aichi, Japan) was inserted through the needle into the intrahepatic bile duct. Both the stomach and bile duct walls were dilated using a mechanical dilator (ES Dilator; Zeon Medical, Tokyo, Japan). After tract dilation, a dedicated plastic stent was placed from the intrahepatic bile duct to the stomach (Fig. 1). In cases where the mechanical dilator could not be inserted into the biliary tract, the catheter for ERCP or a 4mm balloon dilator (REN; Kaneka, Osaka, Japan) was first inserted into the biliary tract, and the guidewire was exchanged for a 0.025-inch guidewire (VisiGlide2; Olympus, Tokyo, Japan). Similarly, in cases where the stent delivery system could not be inserted into the biliary tract, the guidewire was exchanged for a 0.025-inch guidewire.

### 2.4. Statistical analysis

Data are presented as median with range or number with percentage. All statistical analyses were performed using the Bell Curve for Excel (Social Survey Research Information, Tokyo, Japan).

## 3. Results

Fourteen eligible patients (8 men and 6 women) were included in this study. The patients age ranged from 55 to 93 years, with a median age of 76 years. Table 1 presents the background characteristics of all eligible cases. Regarding underlying diseases, there were 8 cases of pancreatic cancer (57.1%), 2 cases of metastatic liver tumor (14.3%), 2 cases of bile duct stones (14.3%), 1 case of hilar cholangiocarcinoma (7.1%), and 1 case of gallbladder cancer (7.1%). Regarding gastrointestinal anatomy, there were 11 cases (78.6%) of normal and 3 cases (21.4%) of gastric resection with Roux-en-Y. The reasons for EUS-HGS were duodenal obstruction in 7 cases (50.0%), surgically altered anatomy in 3 cases (21.4%), and failed ERCP in 4 cases (28.6%). Technical success was achieved in 11 cases (78.6%).

**Table 1:** Baseline patient characteristics of all eligible cases.

	n = 14
Age, year, median (range)	76 (55–93)
Sex, male, n (%)	8 (57.1)
Disease, n (%)	
Pancreatic cancer	8 (57.1)
Metastatic liver tumor	2 (14.3)
Bile duct stones	2 (14.3)
Gallbladder cancer	1 (7.1)
Hilar cholangiocarcinoma	1 (7.1)
Anatomy, n (%)	
Normal	11 (78.6)
Gastric resection with Roux-en-Y	3 (21.4)
Reasons for EUS-HGS, n (%)	
Duodenal obstruction	7 (50.0)
Failed ERCP-related procedure	4 (28.6)
Surgical altered anatomy	3 (21.4)

ERCP = endoscopic retrograde cholangiopancreatography, EUS-HGS = endoscopic ultrasound-guided hepaticogastrostomy.

Table 2 presents the clinical outcomes of the 11 cases of technical success. There were 5 cases of puncturing B2 (45.5%). The puncture bile duct diameter ranged from 3.1 to 5.7 mm with a median of 4.4 mm. EUS-AG was combined with EUS-HGS in 2 cases (18.2%) and clinical success was achieved in all the cases. The procedure time ranged from 15 to 93 minutes, with a median duration of 35 minutes. Regarding the type of stent placed for hepaticogastrostomy, a plastic stent was placed in 10 cases (90.9%) and a metal stent was placed in 1 case (9.1%). Early adverse events occurred in 4 cases (36.4%), all of them developed biliary peritonitis. Late adverse events occurred in 1 case (9.1%), and this was biloma. A change to a 0.025-inch guidewire during the procedure was required in 8 cases (72.7%). Esophageal puncture was not performed.

**Table 2:** Clinical outcomes of 11 successful cases.

	n = 11
Puncture site, n (%)	
B2	5 (45.5)
B3	6 (54.5)
Puncture bile duct diameter, mm, median (range)	4.4 (3.1–5.7)
Combined with ultrasound-guided antegrade procedure, n (%)	2 (18.2)
Clinical success, n (%)	11 (100)
Procedure time, min, median (range)	35 (15–93)
Type of stent placed for hepaticogastrostomy, n (%)	
Plastic stent	10 (90.9)
Metal stent	1 (9.1)
Early adverse events, n (%)	4 (36.4)
Biliary peritonitis	4 (36.4)
Late adverse events, n (%)	1 (9.1)
Biloma	1 (9.1)
Exchange to 0.025-inch guidewire during procedure, n (%)	8 (72.7)
Puncture from esophagus, n (%)	0

## 4. Discussion

This study examined the feasibility of EUS-HGS using a 22-gauge FNA needle. EUS-HGS using a 22-gauge FNA needle combined with a 0.018-inch guidewire was performed in difficult cases using a 19-gauge FNA needle combined with a 0.025-inch guidewire. Although this study included a small number of cases, EUS-HGS using a 22-gauge FNA needle showed a high technical success rate. However, because the mechanical dilator or stent delivery system could not be inserted into the biliary tract in 72.7% of the cases using the 0.018-inch guidewire, the guidewire was exchanged for a 0.025-inch guidewire.

The use of a 0.018-inch guidewire allowed EUS-HGS to be performed using a 22-gauge FNA needle. Compared to a 19-gauge FNA needle, a 22-gauge FNA needle offers more flexibility for manipulation and is superior for bile duct puncture. However, several concerns have been raised regarding the use of 0.018-inch guidewires. These include poor maneuverability, and insufficient force transmission. Due to these disadvantages, EUS-HGS is generally performed with a 0.025-inch guidewire and a 19-gauge FNA needle. However,the device is currently being improved with dedicated dilators for 0.018-inch guidewires being developed.^[9,10]^

There are only a few reports on EUS-HGS using a 0.018-inch guidewire and 22-gauge FNA needle. Iwashita reported that in a study of 26 patients who underwent EUS-BD using a 22-gauge FNA needle combined with a 0.018-inch guidewire, technical success rate was 100%, and the occurrence rate of AEs was 19%.^[9]^ Ogura reported that in a study of 10 patients who underwent EUS-HGS using a 22-gauge FNA needle combined with a 0.018-inch guidewire, the technical success rate of stent placement with only a 0.018-inch guidewire was 87.5%, the overall procedure success rate was 100%, and the occurrence rate of adverse events was 10%.^[5]^

In this study, EUS-HGS was technically successful in 78.6% of cases using a 22-gauge FNA needle combined with a 0.018-inch guidewire initially, even in cases considered difficult with the usual 19-gauge FNA needle combined with a 0.025-inch guidewire. However, in 72.7% of the cases that initially started with a 22-gauge FNA needle combined with a 0.018-inch guidewire, the guidewire was exchanged for a 0.025-inch guidewire as the mechanical dilator or stent delivery system could not be inserted into the biliary tract. This increased the cost of the procedure. EUS-HGS using a 22-gauge FNA needle combined with a 0.018-inch guidewire should be limited to cases of insufficient bile duct dilation. The limitations of this study include its retrospective design and the small number of cases gave only a small sample size.

## 5. Conclusion

Findings suggest EUS-HGS using a 22-gauge FNA needle is initially effective. However, in 72.7% of the cases started using the 0.018-inch guidewire, the guidewire was exchanged for a 0.025-inch guidewire during procedure.

## Acknowledgments

The authors would like to thank the staff involved in performing the EUS-BD procedures at Chiba University Hospital. We would also like to thank editage for the English language review.

## Author contributions

**Data curation:** Koji Takahashi, Hiroshi Ohyama, Mayu Ouchi, Motoyasu Kan, Hiroki Nagashima, Yotaro Iino, Yuko Kusakabe, Kohichiroh Okitsu, Izumi Ohno.

**Formal analysis:** Koji Takahashi.

**Investigation:** Koji Takahashi, Hiroshi Ohyama, Mayu Ouchi, Motoyasu Kan, Hiroki Nagashima, Yotaro Iino, Yuko Kusakabe, Kohichiroh Okitsu, Izumi Ohno.

**Methodology:** Koji Takahashi.

**Project administration:** Koji Takahashi, Hiroshi Ohyama.

**Software:** Koji Takahashi.

**Supervision:** Hiroshi Ohyama, Yuichi Takiguchi, Naoya Kato.

**Writing – original draft:** Koji Takahashi.

**Writing – review & editing:** Koji Takahashi.
